# Hemoglobin A1c in type 2 diabetes mellitus patients with preserved ejection fraction is an independent predictor of left ventricular myocardial deformation and tissue abnormalities

**DOI:** 10.1186/s12872-023-03082-5

**Published:** 2023-01-25

**Authors:** Zhiming Li, Dan Han, Tianfu Qi, Jie Deng, Lili Li, Chao Gao, Wei Gao, Haiyan Chen, Lihua Zhang, Wei Chen

**Affiliations:** 1 Department of Radiology, First Affiliated Hospital of Kunming Medical University, 295 Xichang Road, Kunming, 650032 China; 2 Department of General Medicine, First Affiliated Hospital of Kunming Medical University, 295 Xichang Road, Kunming, 650032 China; 3 Department of Radiology, First People’s Hospital of Honghe State, 1 Xiyuan Road, Honghe, 661100 China

**Keywords:** Type 2 diabetes mellitus, Hypertension, Cardiac magnetic resonance, T1 mapping, Feature tracking, Hemoglobin A1c

## Abstract

**Background:**

Early detection of subclinical myocardial dysfunction in patients with type 2 diabetes mellitus (T2DM) is essential for preventing heart failure. This study aims to search for predictors of left ventricular (LV) myocardial deformation and tissue abnormalities in T2DM patients with preserved ejection fraction by using CMR T1 mapping and feature tracking.

**Methods:**

70 patients and 44 sex- and age-matched controls (Cs) were recruited and underwent CMR examination to obtain LV myocardial extracellular volume fraction (ECV) and global longitudinal strain (GLS). The patients were subdivided into three groups, including 19 normotensive T2DM patients (G1), 19 hypertensive T2DM patients (G2) and 32 hypertensive patients (HT). The baseline biochemical indices were collected before CMR examination.

**Results:**

LV ECV in T2DM patients was significantly higher than that in Cs (30.75 ± 3.65% vs. 26.33 ± 2.81%; *p* < 0.05). LV GLS in T2DM patients reduced compared with that in Cs (−16.51 ± 2.53% vs. −19.66 ± 3.21%, *p* < 0.001). In the subgroup analysis, ECV in G2 increased compared with that in G1 (31.92 ± 3.05% vs. 29.59 ± 3.90%, *p* = 0.032) and that in HT, too (31.92 ± 3.05% vs. 29.22 ± 6.58%, *p* = 0.042). GLS in G2 significantly reduced compared with that in G1 (−15.75 ± 2.29% vs. −17.27 ± 2.57%, *p* < 0.05) and in HT, too (−15.75 ± 2.29% vs. −17.54 ± 3.097%, *p* < 0.05). In T2DM group, including both G1 and G2, hemoglobin A1c (HbA1c) can independently forecast the increase in ECV (β = 0.274, *p* = 0.001) and decrease in GLS (β = 0.383, *p* = 0.018).

**Conclusions:**

T2DM patients with preserved ejection fraction show increased ECV but deteriorated GLS, which may be exacerbated by hypertension of these patients. Hemoglobin A1c is an index that can independently predict T2DM patients’ LV myocardial deformation and tissue abnormalities.

## Background

Diabetes mellitus is a metabolic disorder characterized by insulin insensitivity, insulin deficiency, and impaired biological function caused by genetic and environmental factors. The diabetes atlas of the International Diabetes Federation shows that the global burden of diabetes and its complications are currently increasing [1]. As a common pathway for many pathological mechanisms underlying type 2 diabetes mellitus (T2DM), myocardial fibrosis can lead to heart failure (HF) [2]. HF with preserved ejection fraction (HFpEF; i.e., left ventricular ejection fraction [LVEF] ≥ 50%) is the most common type of HF in patients with T2DM [3]. However, T2DM patients with preserved ejection fraction (EF) do not manifest obvious clinical symptoms. Because hardly any abnormalities of the cardiac structure nor the LVEF can be captured using routine imaging such as echocardiography, the medical community is not aware of the necessity for cardiac protection in clinical treatment. Thus, to optimize intervention plans and prevent HF, the early detection of subclinical myocardial dysfunction in diabetic patients with preserved EF is essential.

As a common concomitant condition in most T2DM patients, hypertension (HT), together with T2DM, contributes to a four-fold increased risk of cardiovascular mortality as compared with normal controls [4]. In addition, the two conditions are associated with structural and functional atrioventricular abnormalities, ultimately resulting in cardiac dysfunction and HF [5]. However, as risky and serious as these conditions are, there has been a scant amount of research on the effect of HT on myocardial fibrosis and cardiac insufficiency in T2DM patients with preserved EF.

It is important to detect subclinical cardiac dysfunction in T2DM patients with preserved EF via noninvasive examination. In recent years, the extracellular volume fraction (ECV) derived from cardiac magnetic resonance (CMR) T1 mapping has been widely employed to detect the expansion of the extracellular matrix (ECM), and it has been considered indicative of the existence and severity of diffuse myocardial fibrosis [6]. In addition, CMR feature tracking with high resolution has been introduced to evaluate myocardial deformation as an indicator of subclinical myocardial dysfunction [7]. Previous studies have reported that the development of cardiac dysfunction and myocardial fibrosis may be associated with hyperglycemia due to the accumulation of glycosylation end products [8]. Under such circumstances, it is particularly important to look for precise and sensitive imaging and serological indicators that can reflect abnormalities of myocardial tissues and cardiac insufficiency in T2DM patients with preserved EF. It is against this background that the present study aims to quantitatively characterize both abnormalities of the myocardial tissue and strain alterations in T2DM patients with preserved EF (LVEF ≥ 50%); to explore the impact of HT on cardiac dysfunction in T2DM patients; and to find indicators that can independently predict myocardial fibrosis and myocardial deformation.

## Methods

### Study population

A cross-sectional study was carried out on 138 hospitalized patients from July 2019 to July 2021. Patients with T2DM were required to be between 30 and 70 years old and to meet the American Diabetes Association standards[9]: having a fasting plasma glucose (FPG) level of ≥ 7.0 mmol/L; a 2-h plasma glucose (PG) level of ≥ 11.1 mmol/L during an oral glucose tolerance test (OGTT); a hemoglobin A1c (HbA1c) level of ≥ 6.5%; or, in a patient with classic symptoms of hyperglycemia or hyperglycemic crisis, a random plasma glucose level of ≥ 11.1 mmol/L. HT was defined by either a systolic blood pressure (SBP) level of ≥ 140 mmHg, a diastolic blood pressure of (DBP) ≥ 90 mmHg, or both, or by the patient receiving current treatment for HT according to the global HT practice guidelines issued by the International Society of Hypertension in 2020 [10]. Patients who underwent CMR for chest pain, chest tightness, and palpitations but who did not show abnormalities as a result of CMR, electrocardiography, and myocardial enzyme testing served as the control group. Key exclusion criteria included the following: (1) contraindications for CMR; (2) a history of myocardial infarction or other organic heart disease; (3) atrial fibrillation; (4) LVEF < 50%; (5) congestive HF; and (6) conditions affecting the accuracy and reliability of the study measurements (e.g., arrhythmia affecting cardiac gating, the inability to hold the breath long enough during CMR scanning).

### Anthropometric and biochemistry measurements

Baseline demographic and clinical characteristics were recorded 1–7 days before CMR examination. The hematocrit level (HCT) was measured to calculate the ECV of the left ventricular (LV) myocardium. Other biochemical indices were collected, including FPG, glycosylated serum protein (GSP), HbA1c, total cholesterol (TC), free cholesterol (F-CHO), triglycerides (TG), high-density lipoprotein cholesterol (HDL-C), and low-density lipoprotein cholesterol (LDL-C).

### Cardiac magnetic resonance scanning protocol

CMR imaging was performed using a 1.5-T MR scanner (MAGNETOM Amira, Siemens Medical Systems, Germany) with vector-electrocardiographic gating and an 18-channel phased array cardiac surface coil. The cardiovascular imaging protocol consisted of cine steady-state free precession sequences in the LV long axis (two-, three-, and four-chamber views) and short axis (coverage from the base to the apex segment) to evaluate cardiac function and structure. Pre- and post-contrast T1 electrocardiogram-gated steady-state free-precession-based modified Look-Locker inversion recovery technique with a 5(3)3 pattern (i.e., acquiring 5 images after the first inversion, followed by a 3 heartbeat pause and then acquiring 3 images after the second inversion) was used to evaluate diffuse myocardial fibrosis (i.e., increased CMR-ECV). Late gadolinium enhancement (LGE) sequences obtained 10 min after gadolinium injection (0.2 mmol/kg; Gadovist, Bayer Healthcare, Berlin, Germany) were used to assess myocardial focal fibrosis [11].

Native and post-contrast T1 mapping using modified Look-Locker inversion recovery technique sequences were obtained on the apex, mid, and basal short-axis planes of the LV before and 12–15 min after the application of contrast agent using the following parameters: TR/TE 346.56/1.12 ms; 8 mm thickness; field of view of 360 mm^2^ × 300 mm^2^; matrix size of 256 × 166; flip angle of 35°; and PAT factor of 2. T1 maps were generated immediately after the scan using a motion correction technique.

All of the CMR scanning protocols followed the guidelines recommended by the Society for Cardiovascular Magnetic Resonance [12].

### Assessment of cardiac volume and function

Cardiac structural and functional parameters were assessed using cine images offline by CVI42, a commercial post-processing software (Circle Cardiovascular Imaging, Calgary, Alberta, Canada). The endocardial and epicardial traces were manually delineated in the serial short-axis slices in the end-diastolic and end-systolic phases, respectively. The moderator bands and papillary muscles were excluded from the LV. The LV functional parameters—including LV end-diastolic volume (LVEDV), LV end-systolic volume (LVESV), LVEF and LV mass (LVM)—, were automatically acquired. All of the cardiac functional parameters were indexed to the body surface area (BSA).

### Cardiac magnetic resonance T1 mapping

Using the CVI42 software, the myocardium of the LV (inner and outer contour) was segmented into the basal, mid, and apical ventricular slices of the short-axis images in accordance with recommendations that omitted the influence of the surrounding fat or blood. Segments affected by apparent artifacts or the presence of LGE were omitted to avoid distorting the measured values [6, 13]. The LV myocardium was divided into 17 segments according to the American Heart Association classification. The measurements were obtained for segments 1 through 16 at the basal, mid, and apex-ventricular levels in the acquired short-axis images [14]. Another region of interest was located in the blood volume. The ECV was derived using the following formula [15]:

$$ECV = \left( {1 - HCT} \right) \times \left[ {\left( {1/{ }T1_{MyoPost} - { }1/T1_{MyoPre} } \right)/\left( {1/T1_{BloodPost} - 1/T1_{BloodPre} } \right)} \right]$$

**Fig. 1:**
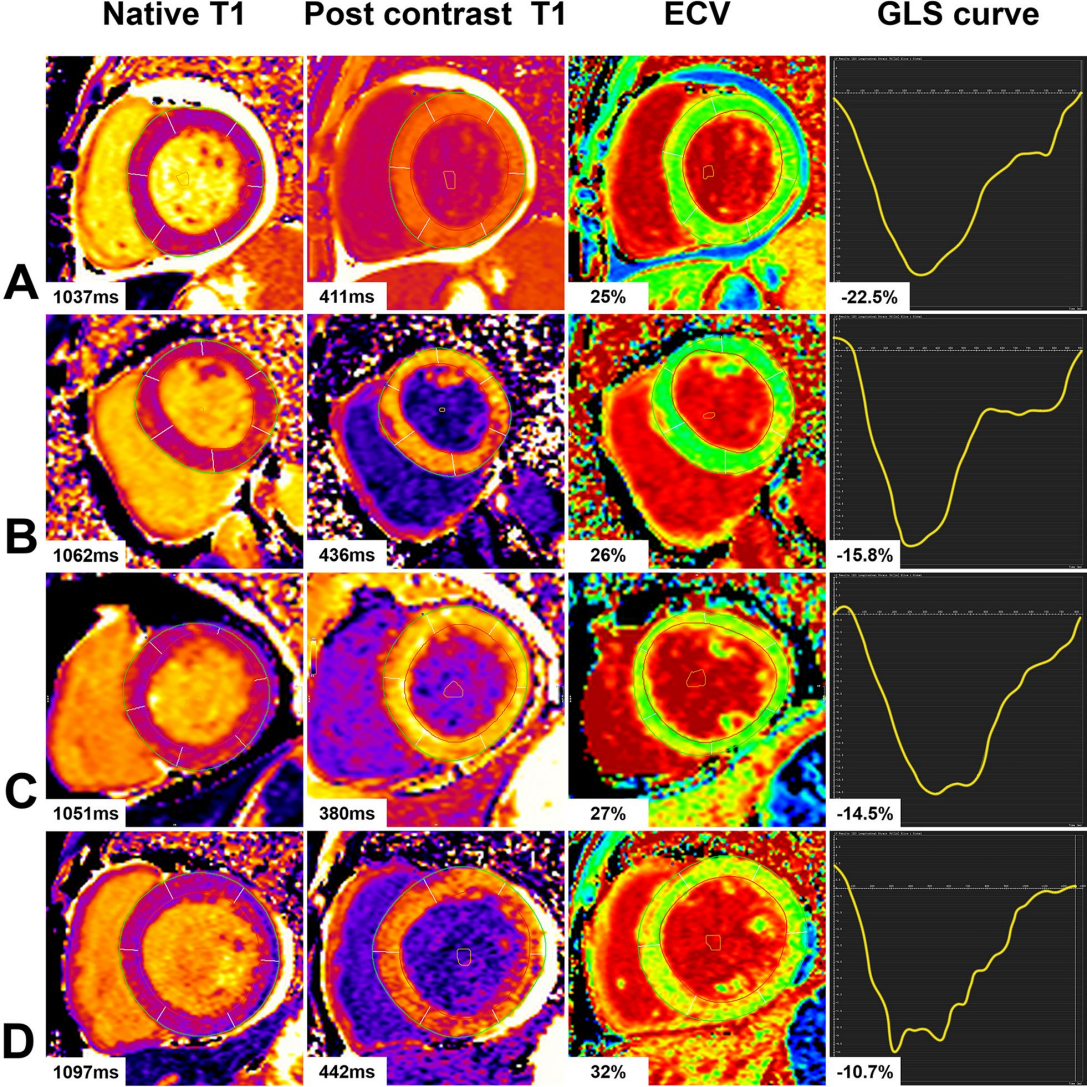
T1 maps and GLS curve of typical cases in each group. A represents the Native T1 = 1037 ms, Post contrast T1 = 411 ms, ECV = 25%, and GLS = -22.5% of a control subject; B represents the Native T1 = 1062 ms, Post contrast T1 = 436 ms, ECV = 26%, and GLS = -15.8% of a hypertensive patient; C represents the Native T1 = 1051 ms, Post contrast T1 = 380 ms, ECV = 27%, and GLS = -14.5% of a normotension T2DM patient; D represents the Native T1 = 1097 ms, Post contrast T1 = 442 ms, ECV = 32%, and GLS = -10.7% of a hypertensive T2DM patient

T1_MyoPre_, T1_MyoPost_, T1_BloodPre_, and T1_BloodPost_ were the T1 measurements obtained from the myocardium and from the blood before and after the contrast enhancement, respectively (Fig. 1). The hematocrit level was obtained via routine hematological examination of the patients 1 to 7 days before CMR scanning.

### Cardiac magnetic resonance feature tracking

Global measurements of LV deformation—including global radial strain (GRS), global circumferential strain (GCS), and global longitudinal strain (GLS)—were analyzed using the CVI42 software’s CMR feature tracking in accordance with the parameters of previous studies [16]. LV endocardial and epicardial borders were manually traced at the end of diastole in electrocardiogram-gated steady-state free precession four-, three- and two-chamber long-axis sequences using a point-and-click approach. The automatic border tracking algorithm of the software was employed to track image features throughout the cardiac cycle. The tracking was visually reviewed, and, if necessary, manually corrected through border adjustment. GRS and GCS were measured in the short axial field of view, and GLS measurements were obtained in two-, three-, and four-chamber views.

### Statistical analysis

The normality of all continuous data was tested using the Kolmogorov–Smirnov test. Normally distributed data were expressed as mean ± standard deviation, nonparametric data were given as the median and the interquartile range, and categorical variables were displayed as the number of subjects and their percentages. Categorical variables were analyzed with the chi-squared test. The differences between the two groups were analyzed with the independent-sample Student’s t-test for continuous variables and with the Mann–Whitney U test for nonparametric continuous variables. Analysis of variance was used to assess the differences across the three groups. Correlational analyses were performed with Pearson’s correlation for normally distributed variables, whereas Spearman’s correlation was performed for non-normally distributed variables. Multivariable linear regression analysis was performed to identify the independent predictors of diffuse myocardial fibrosis and myocardial deformation. All of the candidate variables (*p* < 0.05 for the univariate linear regression analysis and without collinearity), including SBP, FPG, HbA1c, were selected for entry into the multiple linear stepwise regression model. All of the comparisons and correlations were two-tailed, with *p* values of less than 0.05 considered statistically significant. All of the data processing and statistical analyses were performed using SPSS for Windows (version 20.0; IBM SPSS Statistics, Chicago, IL, USA).

## Results

### Clinical characterization

For this study, 138 subjects were recruited from July 2019 through July 2021. Of these 138, 14 and 10 were excluded due to LVEF <50% and poor image quality, respectively. In the end, 114 subjects were enrolled and divided into the T2DM (n = 38), HT (n = 32), and control (n = 44) groups (Table 1). T2DM patients were then divided into two subgroups: normotensive T2DM (G1; n = 19) and hypertensive T2DM (G2; n = 19). T2DM patients had significantly higher body mass index (BMI), BSA, SBP, FPG, GSP, and HbA1c values as compared with controls. They also had lower high-density lipoprotein cholesterol values (*p* < 0.05) than controls. In addition, G2 and HT patients also had higher body mass index (BMI), SBP, and DBP values than controls. FPG, GSP, and HbA1c levels in both G1 and G2 patients were higher than those of both the control group and the HT group (see Table 1).

**Table 1:** Clinical characteristics of subjects

	Cs (n = 44)	T2DM (n = 38)	G1 (n = 19)	G2 (n = 19)	HT (n = 32)
Age/year	49 ± 11	53 ± 11	52 ± 11	54 ± 12	48 ± 12
Male/ (n)/%	27 (61)	22 (63)	10 (59)	12 (66)	20 (62)
Height/ (m)	1.66 ± 0.09	1.68 ± 0.08	1.66 ± 0.08	1.65 ± 0.08	1.65 ± 0.07
Weight/ (kg)	60.00 ( 55.25, 72.00)	70.00 (64.50, 78.00)^a^	70.00 (60.50, 34.50)	71.00 (66.00, 89.00)^**a**^	71.00 (65.25, 79.50)^a^
BMI/ (kg/m^2^)	22.94 (20.00, 25.52)	25.51 (23.08, 28.16)^a^	24.09 (21.71, 27. 62)	27.06 (24.13, 29.40)^a, b^	25.88 (24.09, 27.70)^a^
BSA/m^2^	1.70 ± 0.18	1.86 ± 0.27^a^	1.83 ± 0.3^a^	1.88 ± 0.24^a^	1.82 ± 0.14^a^
SBP/mmHg	118.36 ± 10.80	133.08 ± 14.65^a^	124.82 ± 11.03	140.88 ± 13.49^a, b^	139.96 ± 29.87^a, b^
DBP/mmHg	76.93 ± 9.33	82.60 ± 12.42^a^	76.64 ± 10.16	88.22 ± 11.93^a, b^	90.62 ± 22.89^a, b^
HR/bpm	76 ± 13	71 ± 12	69 ± 10	73 ± 13	76 ± 14
Diabetes duration/y	–	6.65 ± 4.49	6.41 ± 3.35	6.88 ± 5.45	–
HCT /%	43.77 ± 3.73	42.77 ± 4.80	41.35 ± 4.32	44.11 ± 4.96	42.66 ± 4.92
hemoglobin/ (g/L)	148.00 ± 15.10	142.94 ± 15.99	139.29 ± 17.05	146.38 ± 14.55	145.43 ± 18.54
GSP/ (mmol/L)	278.70 ± 49.03	338.19 ± 71.90^a^	333.59 ± 63.16^a^	43.13 ± 82.92^a^	260.75 ± 21.58^b, c^
HbA1C/%	5.57 ± 0.33	8.09 ± 1.86^a^	8.50 ± 2.13^a^	7.65 ± 1.49^a^	5.75 ± 0.43^b, c^
FPG (mmol/L)	4.94 (4.57, 5.24)	7.11 (6.13, 9.13)^a^	6.67 (5.73, 9.76)^a^	7.27 (6.39, 8.61)^a^	5.14 (4.67, 5.72)^b, c^
TC/ (mmol/L)	4.34 ± 0.89	4.51 ± 1.05	4.54 ± 1.15	4.49 ± 0.97	4.44 ± 1.00
F-CHO/ (mmol/L)	1.49 ± 0.36	1.63 ± 0.44	1.56 ± 0.38	1.69 ± 0.49	1.55 ± 0.38
LDL-C/ (mmol/L)	2.49 ± 0.72	2.71 ± 0.92	2.88 ± 0.95	2.54 ± 0.88	2.65 ± 0.77
HDL-C/ (mmol/L)	1.23 ± 0.41	0.98 ± 0.21^a^	0.95 ± 0.19^a^	1.02 ± 0.23^a^	1.03 ± 0.20^a^
TG/ (mmol/L)	1.20 (0.84, 2.26)	1.61 (1.30, 2.38)	1.81 ± 0.91	2.11 ± 1.44	1.67 (1.26, 2.39)

*BMI*  body mass index, *BSA*  body surface area, *SBP*  systolic blood pressure, *DBP*  diastolic blood pressure, *HR*  heart rate, *HCT*  hematocrit, *GSP*  glycosylated Serum Protein, *HbA1c*  hemoglobin A1c, *FPG*  fasting plasma glucose, *TC*  total cholesterol, *F-CHO*  free cholesterol, *LDL-C*  low density lipoprotein cholesterol, *HDL-C*  high-density lipoprotein cholesterol, *TG*  triglycerides

All results shown as percent, Mean ± SD or median (25th, 75th percentiles)

^a^*p* value versus Cs, *p* < 0.05

^b^*p* value versus G1, *p* < 0.05

^c^*p* value G2, *p* < 0.05

*p* < 0.05 is considered to indicate statistical significance

### Cardiac magnetic resonance structural and functional characterization

No subendocardial delayed enhancement (myocardial ischemia/infarction) or significant subepicardial or middle myocardial delayed enhancement was observed in any of the subjects. T2DM patients exhibited significantly higher LVM and LVM index values as compared with controls (*p* < 0.001). The LVM and LVM index values of the G2 and HT groups were also significantly higher than those of the controls (*p* < 0.001). However, the G1 and G2 groups did not differ from each other with regard to their LVM and LVM index values. There was no significant difference between T2DM patients and controls with regard to their LVEF values (61.77 ± 13.69 vs. 60.27 ± 6.21; *p* = 0.55). There were no significant differences in LVEF values among the G1, G2, and HT groups, either (all *p* > 0.05). No significant differences were observed in other functional parameters between the patients and the controls (see Table 2).

**Table 2:** Cardiac magnetic resonance characteristics of subjects

	Cs (n = 44)	T2DM (n = 38)	G1 (n = 19)	G2 (n = 19)	HT (n = 32)
LVM/g	79.05 (64.67, 101.61)	117.05 (87.84, 172.50)^a^	95.60 (79.90, 172.50)	130.17 (88.80, 217.23)^a^	120.00 (94.27, 141.77)^a^
LVM index/(g/m^2^)	49.26 (39.85, 58.00)	62.68 (48.92, 88.77)^a^	54.32 (44.95, 91.41)^a^	68.79 (50.12, 96.97)^a^	67.30 (56.62, 77.87)^a^
LVEDV/ml	129.45 (109.47, 144.42)	136.68 (108.49, 167.64)^a^	135.30 (105.30, 148.51)	159.50 (123.65, 178.23)^a^	130.84 (110.95, 150.47)
LVESV/ml	52.76 (41.80, 63.12)	47.74 (33.42, 72.50)	43.80 (30.05, 59.66)	55.65 (33.73, 91.61)	49.70 (42.75, 64.30)
LVSV/ml	77.41 ± 15.00	90.22 ± 28.96^a^	89.00 ± 34.75	91.38 ± 23.19^a^	79.57 ± 19.66
LVEDVi/(ml/m^2^)	75.55 (68.62, 82.75)	75.65 (65.85, 83.88)	71.61 (65.85, 81.54)	78.93 (63.26, 95.42)	71.70 (60.82, 84.75)
LVESVi/(ml/m^2^)	31.25 (26.60, 35.60)	27.50 (18.66, 39.77)	26.63 (17.42, 36.04)	30.23 (18.78, 44.90)	26.90 (19.65, 33.11)
LVSVi/ (ml/m^2^)	45.98 ± 9.46	48.37 ± 13.45	48.77 ± 15.20	47.99 ± 12.00	43.52 ± 10.29
LVEF/%	60.27 ± 6.21	61.77 ± 13.69	63.25 ± 13.85	60.38 ± 13.79	61.15 ± 10.47
LVGRS/%	33.15 ± 8.01	32.41 ± 6.00	32.60 ± 6.75	32.22 ± 5.36	32.37 ± 4.56
LVGCS/%	−23.74 ± 3.31	−23.24 ± 3.25	−23.14 ± 3.01	−23.34 ± 3.49	−22.66 ± 3.00
LVGLS/%	−19.66 ± 3.21	−16.51 ± 2.52^a^	−17.27 ± 2.57^a^	−15.75 ± 2.29^a, b^	−17.54 ± 3.09^a, c^
Apex native T1/ms	1027.53 ± 33.58	1027.36 ± 48.08	1025.00 ± 55.34	1029.87 ± 40.65	1025.28 ± 48.97
Mid native T1/ms	1031.54 ± 28.96	1052.26 ± 39.67^a^	1046.70 ± 47.89	1057.82 ± 29.77^a^	1060.90 ± 41.37^a^
Base native T1/ms	1037.00 ± 26.35	1062.78 ± 44.08^a^	1067.56 ± 55.63	1058.00 ± 29.53^a^	1070.31 ± 42.00^a^
Global native T1/ms	1035.02 ± 26.65	1060.17 ± 43.63^a^	1051.64 ± 46.65	1068.70 ± 39.93^a^	1059.46 ± 34.74^a^
Apex post contrast T1/ms	446.65 ± 61.12	443.30 ± 51.47	443.05 ± 62.06	443.56 ± 39.29	439.40 ± 58.76
Mid post contrast T1/ms	460.47 ± 58.28	453.76 ± 45.07	456.23 ± 50.98	451.29 ± 39.71	458.87 ± 58.74
Base post contrast T1/ms	465.84 ± 58.20	457.50 ± 50.20	462.31 ± 57.62	452.68 ± 42.87	459.28 ± 58.55
Global post contrast T1/ms	456.25 ± 59.64	446.50 ± 41.14	452.29 ± 47.12	440.70 ± 34.63	438.03 ± 67.95
Apex ECV/%	26.60 ± 2.63	28.76 ± 2.10^a^	28.81 ± 2.46^a^	28.71 ± 1.77^a^	27.76 ± 2.49
Mid ECV/%	25.78 ± 2.35	29.70 ± 3.18^a^	28.99 ± 3.78^a^	30.41 ± 2.35^a, b^	28.12 ± 2.03^a, c^
Base ECV/%	25.93 ± 2.43	30.03 ± 3.30^a^	28.43 ± 3.33^a^	31.63 ± 2.44^a, b^	28.53 ± 2.48^a, c^
Global ECV/%	26.33 ± 2.81	30.75 ± 3.65^a^	29.59 ± 3.90^a^	31.92 ± 3.05^a, b^	29.22 ± 6.58^a, c^

*LVM*  left ventricular mass, *LVEDV*  left ventricular end diastolic volume, *LVESV*  left ventricular end systolic volume, *LVSV*  left ventricular stroke volume, *LVEDVi*  left ventricular end diastolic volume index, *LVESVi*  left ventricular end systolic volume index, *LVSVi*  left ventricular stroke volume index, *LVEF*  left ventricular ejection fraction, *LVGLS*  left ventricular global longitudinal strain, *LVGRS*  left ventricular global radial strain, *LVGCS*  left ventricular global circumferential strain, *ECV*  extracellular volume fraction

All results shown as percent, Mean ± SD or median (25th, 75th percentiles)

^a^*p* value versus Cs, *p* < 0.05

^b^*p* value versus G1, *p* < 0.05

^c^*p* value G2, *p* < 0.05

*p* < 0.05 is considered to indicate statistical significance

### Cardiac magnetic resonance T1 mapping characterization

**Fig. 2:**
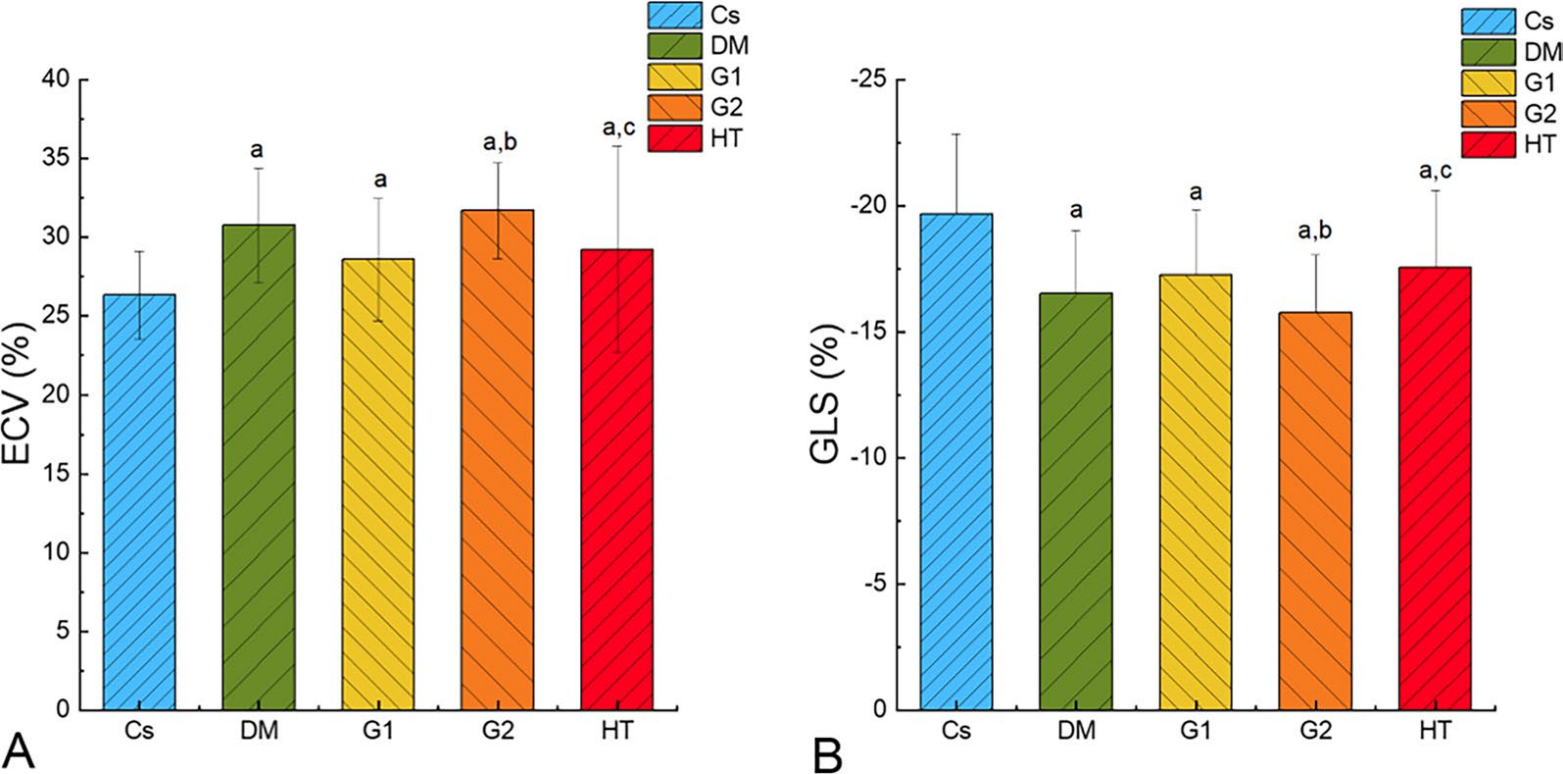
Histogram of ECV and GLS mean values for each group. A showed that ECV in the diabetic group, G1, G2 and HT groups was significantly higher than that in Cs, and ECV in G2 was significantly higher than that in G1 and HT group. B showed that GLS in the diabetic group, G1, G2 and HT groups was significantly lower than that in Cs, and GLS in G2 was significantly lower than that in G1 and HT group

LV global native T1 and ECV values were significantly higher in T2DM patients than in controls (native T1: 1035.02 ms ± 26.65 ms vs. 1060.17 ms ± 43.63 ms, *p* = 0.005; ECV: 26.33% ± 2.81% vs. 30.75% ± 3.65%, *p* < 0.001). In addition, ECV values in the G1, G2, and HT patients (29.59% ± 3.90%, 31.92% ± 3.05%, and 29.22% ± 6.58%, respectively) were significantly higher than those of controls (all *p* < 0.05). G2 patients had statistically significantly higher ECV values than both the G1 (*p* = 0.032) and the HT patients (*p* = 0.042) (Fig. 2). Nevertheless, there was no statistical difference for post contrast T1values across the G1 and G2 patients (see Table 2).

### Cardiac magnetic resonance left ventricular strain characterization

GLS was significantly decreased among T2DM patients as compared with controls (–16.51% ± 2.53% vs. –19.66% ± 3.21%, *p* < 0.001). LV myocardial deformation assessment revealed no significant difference in GCS and GRS values between T2DM patients and controls.

A comparison across the G1, G2, and HT patients showed that GLS was significantly decreased in the G2 patients (–15.75% ± 2.29%) as compared with the G1 (–17.27% ± 2.57%) and the HT patients (–17.54% ± 3.09%) (all *p* < 0.05; see Fig. 2). No significant difference was observed in either GRS or GCS values across the three groups (see Table 2).

### Clinical factors associated with global longitudinal strain and extracellular volume fraction in patients with type 2 diabetes mellitus

The univariate correlation coefficients between the LV ECV, GLS values, and the characterizations of CMR, clinical indexes of the T2DM patients are summarized in Table 3.

**Table 3:** Correlation analysis of ECV, GLS and other clinical and imaging indicators

	ECV		GLS	
	R value	p value	R value	*p* value
Age/y	0.128	0.471	0.108	0.345
Male/(n)/%	0.204	0.253	0.154	0.272
BMI/(kg/m^2^)*	0.152	0.184	0.296	0.009
BSA/m^2^	0.069	0.550	0.115	0.317
SBP/mmHg	0.360	0.001	0.430	< 0.001
DBP/mmHg	0.166	0.146	0.177	0.122
HR/bpm	−0.032	0.778	−0.047	0.686
Diabetes duration/y	0.344	0.463	−0.338	0.516
HCT /%	−0.191	0.094	0.062	0.588
hemoglobin/(g/L)	−0.209	0.068	0.021	0.858
GSP/(mmol/L)	0.258	0.161	0.413	0.021
HbA1c/%	0.447	0.012	0.362	0.046
FPG/(mmol/L)*	0.358	0.004	0.423	0.001
TC/(mmol/L)	−0.063	0.628	0.182	0.160
F-CHO/(mmol/L)	0.052	0.690	0.228	0.078
LDL-C/(mmol/L)	−0.088	0.499	0.186	0.150
HDL-C/(mmol/L)	−0.109	0.403	−0.043	0.744
TG/(mmol/L)*	−0.006	0.966	0.049	0.713
LVM index/(g/m^2^)*	0.093	0.417	0.275	0.015
LVEDVi/(ml/m^2^)*	−0.014	0.906	0.040	0.731
LVESVi/(ml/m^2^)*	−0.005	0.965	0.070	0.542
LVEF/%	−0.122	0.286	−0.110	0.338
LVGRS/%	0.014	0.901	0.004	0.969
LVGCS/%	0.082	0.476	−0.019	0.871
LVGLS/%	0.274	0.015		
Global Native T1/ms*	0.477	< 0.001	0.181	0.113
Global Post T1/ms	−0.008	0.947	−0.155	0.176
Global ECV/%			0.274	0.015

*These data were analyzed using Spearman’s correlation, all other data were analyzed using Pearson’s correlation

*p* < 0.05 is considered to indicate statistical significance

**Fig. 3:**
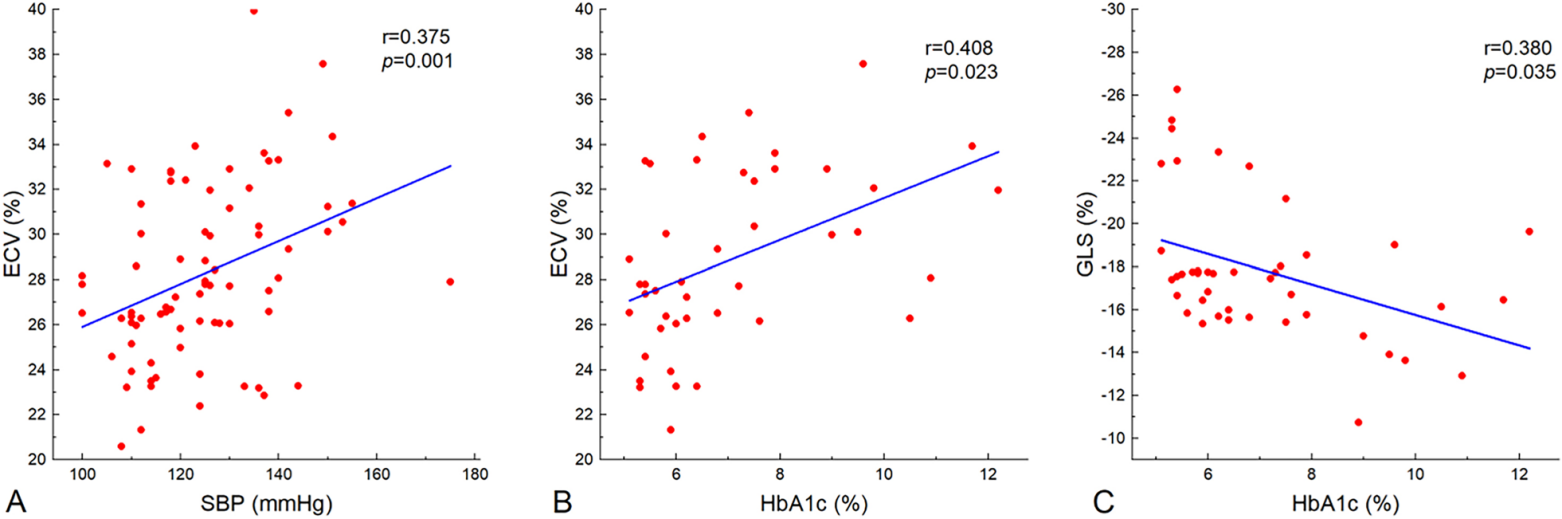
Correlation scatter diagram. ECV was positively correlated with SBP (**A**) and HbA1c (**B**) in T2DM patients. GLS was negatively correlated with HbA1c (**C**) in T2DM patients

Even though the ECV values of the T2DM patients were closely correlated with the SBP, HbA1c, FPG, and GLS values (Fig. 3), no significant associations were detected between the ECV values and the other clinical and CMR parameters (e.g., BMI, LVEDVI, LVEF). Since the variable FPG is a set of non-normally distributed measurement data, we take the log and normalize them before running the regression analysis. Multivariable linear stepwise analysis indicates that HbA1c (β = 0.274; *p* = 0.001) and SBP (β = 0.037; *p* = 0.027) can be used to forecast the increased ECV value independently (model R^2^ = 0.318; Table 4).

**Table 4:** multivariate regression analysis for ECV and GLS in T2DM patients

	Unstandardized β	Standardized β	*P* value
*ECV*			
SBP/mmHg	0.086	0.037	0.027
HbA1C/%	0.947	0.274	0.001
*GLS*			
HbA1C/%	0.585	0.383	0.018

*p* < 0.05 is considered to indicate statistical significance

Despite the fact that the GLS values of the T2DM patients were significantly associated with the SBP, GSP, HbA1c, FPG, LVM index, and ECV values (Fig. 3), no significant association was observed between the GLS value and the other clinical and CMR parameters. Multivariable linear stepwise regression analysis result indicated that the HbA1c value alone (β = 0.383; *p* = 0.018) may suffice to predict the existence of decreased GLS (model R^2^ = 0.123; Table 4).

### ROC curve analysis

**Fig. 4:**
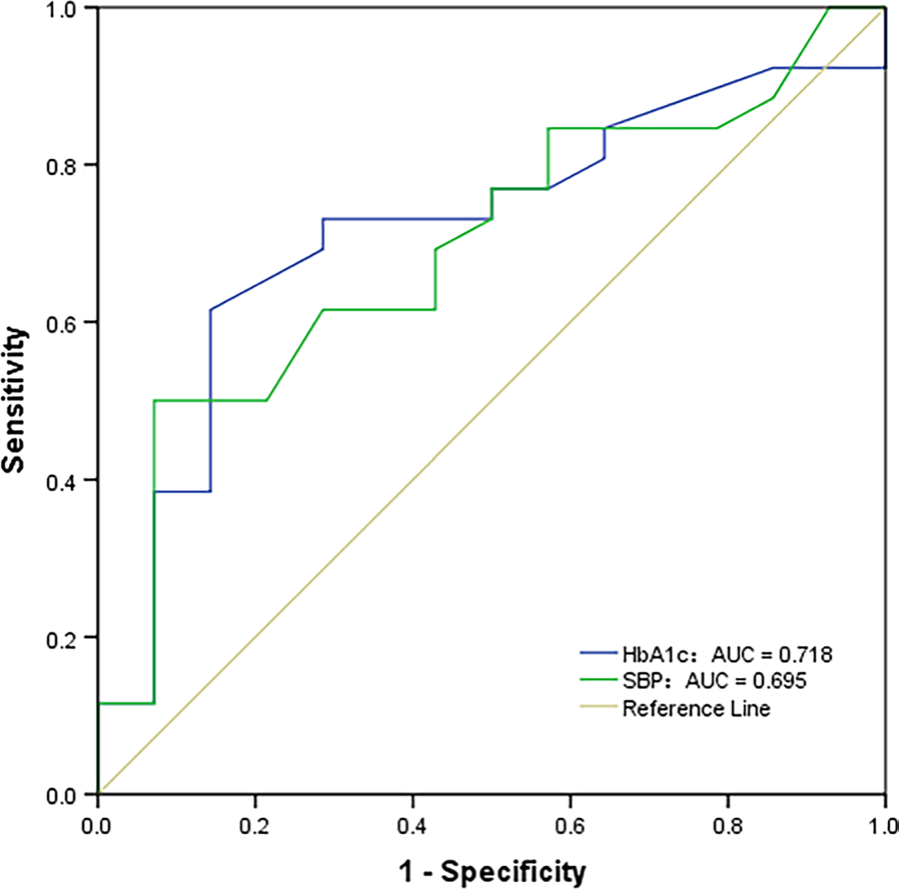
ROC analysis. the sensitivity and specificity of HbA1c (HbA1c > 6.0%) for predicting myocardial fibrosis in T2DM patients were 73.1 and 71.4%. the sensitivity and specificity of SBP (SBP > 133 mmHg) for predicting myocardial fibrosis in T2DM patients were 50.0 and 92.9%

In our study, according to the mean ECV value of the control group (26.33%), we divided patients into non-myocardial fibrosis and myocardial fibrosis. ROC analysis showed that 6.0% was the optimal cutoff values of HbA1c that predicted the risk of myocardial fibrosis (sensitivity 73.1%, specificity 71.4%, and area under the curve (AUC):0.718, *p* = 0.024). The Youden index of the HbA1c was 0.445. And 133 mmHg was the optimal cutoff values of SBP that predicted the risk of myocardial fibrosis (sensitivity 50.0%, specificity 92.9%, and AUC: 0.695, *p* = 0.044). The Youden index of the HbA1c was 0.429 (Fig. 4).

## Discussion

In this study, we applied noninvasive CMR T1 mapping and feature tracking to search for predictors of LV myocardial deformation and tissue abnormalities in T2DM patients with preserved EF. The study demonstrates that T2DM patients and even those with preserved EF developed diffuse myocardial fibrosis and deformation abnormalities: (1) the T2DM group (especially hypertensive T2DM patients) showed significantly increased ECV and deteriorated LV strain; and (2) the increased ECV and injured GLS were affected by an elevated HbA1c, which is a classic metric of poor glycemic control. Therefore, we may comfortably suggest that elevated HbA1c can be employed as a predicator of diffuse myocardial fibrosis and injured myocardial deformation.

Myocardial fibrosis is an important pathophysiological mechanism underlying structural and functional cardiac abnormalities in T2DM patients, and it has been reported to be associated with hyperglycemic metabolism [17, 18]. In the setting of T2DM, hypermetabolism and increased glucose residues work together to promote the production of advanced glycation end products [19]. Because the latter may crosslink ECM proteins and impair ECM degradation by matrix metalloproteinases, it may in turn increase cardiac stiffness, which manifests itself as early diastolic dysfunction [20].

ECV, as a marker of myocardial tissue remodeling, is a physiologically intuitive unit of measurement. An increased ECV, which is more often related to excessive collagen deposition, is a more robust measurement of diffuse myocardial fibrosis [15]. Various studies have demonstrated that myocardial ECV is significantly correlated with ECM (in cases of diffuse myocardial fibrosis) [21, 22]. Therefore, it follows that an increase in ECV in T2DM patients with preserved EF may be associated with diffuse myocardial fibrosis. This finding is in line with previous reports of T2DM patients exhibiting increased myocardial ECV as compared with healthy subjects [23], despite the claim by Levelt and colleagues that the difference in the myocardial ECV between middle-aged T2DM patients and healthy subjects was not significant [24]. Discrepancies like this between studies may be ascribed to the varied durations and types of diabetes, the ages of the patients studied, comorbidities, complications, and medical therapies in different studies and under different experimental conditions.

The biological factor that determines an increase in native T1 is whether there is an increase in the interstitial space (e.g., fibrosis of cardiomyopathy) [15]. This study detected such an increase in native T1 among T2DM patients with preserved EF, which corroborates two previous studies [8, 13]. In the subgroup analysis, although there was no significant difference in native T1 between normotensive T2DM patients and controls, myocardial ECV in these patients was higher than in the control group. This difference may be ascribed to the fact that native T1 is a composite signal of myocytes and ECV with the potential to pseudo-normalize abnormal values. Alternatively, ECV is a physiological parameter derived from the ratio of T1 signal values. ECV may therefore be more reproducible using varied field strengths and acquisition techniques as compared with both native and post-contrast T1 [25]. ECV measures also exhibit better agreement with histological measurements of the collagen volume fraction as compared with isolated postcontrast T1 [26].

In recent years, CMR feature tracking imaging based on routine cine CMR images has emerged as a new technology that can quantitatively characterize myocardial systolic and diastolic function [27, 28]. In this study, we detected impaired LV GLS in T2DM patients with preserved EF as compared with control subjects. The decreased LV GLS in T2DM patients seemed to precede overt LV dysfunction, and it was recognized even in T2DM patients without impaired LVEF. Studies using ultrasound speckle tracking [29, 30] or CMR feature tracking [16] have suggested that diabetic patients exhibit decreases in the longitudinal, circumferential or radial strains as compared with control groups. At the same time, previous studies have demonstrated that diabetic patients and healthy individuals have had similar LV GLS, GRS, GCS and the strain rates [23]. The differences noted in the myocardial strain measurements may be related to differences in study populations and different methods of strain acquisition. There are two possible mechanisms that may help explain why LV global strain in the T2DM group was significantly lower than that of the control group. First, T2DM can lead to LV fibrosis, which in turn can lead to decreased LV compliance[20] and ultimately to impaired myocardial strain. Second, myocardial inflammation in T2DM patients may trigger ventricular remodeling [31]. Due to its good reproducibility, GLS has been used to detect early subclinical myocardial dysfunction in a broad spectrum of cardiac conditions [32]. In this study, in the T2DM patients, only GLS was impaired, whereas GRS and GCS showed no differences when compared with the control group; this may be related to the higher sensitivity of GLS for the evaluation of subclinical cardiac dysfunction as compared with GRS and GCS [33].

Moreover, the present findings demonstrate that hypertensive T2DM patients have higher ECV values as compared with normotensive T2DM patients. Therefore, we tentatively hypothesize that HT can exacerbate the expansion of diffuse myocardial fibrosis in diabetic patients. This hypothesis corroborates the findings of Kwiecinski and colleagues that increased blood pressure levels resulted in higher indices of diffuse myocardial fibrosis (ECV increased) while, at the same time, LVEF fell [34]. Wang and colleagues also reported that diffuse myocardial fibrosis may develop in the presence of hypertensive cardiomyopathy before conventional MRI-detectable LGE occurs. ECV can be employed to identify diffuse myocardial fibrosis at an early stage among hypertensive patients. In addition, elevated ECV is associated with both decreased LV global function and LV remodeling in patients with HT [35]. Bendiab and colleagues investigated the impact of concomitant T2DM on subclinical cardiac function in a cohort of hypertensive patients and found that LV geometry and subclinical LV function as assessed with GLS were more impaired in hypertensive patients with T2DM than in those without T2DM; after adjustment for BP and BMI, T2DM remained an independent predictor of GLS decline (odds ratio = 2.26; 95% confidence interval, 1.11–4.61; *p* = 0.023) [36], which is consistent with the findings of our study.

Results of the present study indicate that poor glycemic control was correlated with diffuse myocardial fibrosis and myocardial strain injury. The systemic hyperglycemia, hyperlipidemia, and inflammation associated with T2DM contribute to the development of cardiac hypertrophy and fibrosis, which in turn increase myocardial stiffness and result in cardiac dysfunction [37]. HbA1c, which reflects the patient’s control of their blood glucose level during the preceding two to three months, has better timeliness and is thus a better predictor of prognosis following coronary heart disease than fasting or admission glucose [9]. A previous study found that a high HbA1c level is more likely to cause damage to cardiomyocytes, which is a powerful predictor of myocardial fibrosis [8]. Another study found that the HbA1c level was negatively correlated with systolic strain or diastolic function in T2DM patients [38]. In the present study, the HbA1c level was found to be associated with ECV and GLS values, and can effectively predict myocardial fibrosis and myocardial strain injury in T2DM with preserved ejection fraction, suggesting that chronic hyperglycemia has an adverse effect on diffuse myocardial fibrosis and strain. We may suggest that, even if the LVEF of a T2DM patient is preserved, the possibility of damage to cardiomyocytes or structure remodeling cannot be ruled out. However, this study show that HbA1c has limited ability to predict T2DM fibrosis. The reason may be that the mechanism of myocardial fibrosis is complex and cannot be fully explained by the change of a single index (HbA1c). On the other hand, the fact that the small sample size of T2DM in this study also has a certain impact on the research results. To sum up, the HbA1c value effectively reflects blood glucose control, and it may also be used to predict myocardial fibrosis and dysfunction. Hence, diabetic patients should monitor their HbA1c levels closely and regularly.

The present study has several limitations. First, it involves a small sample size, although it did meet the minimum sample requirements. Second, the increased myocardial ECV in T2DM patients was not confirmed by pathological analysis due to endomyocardial biopsy being invasive and thus not suitable for the subjects in our study. Previous histological studies have demonstrated that the myocardial ECV exhibits significant correlation with the myocardial collagen volume fraction [39], which confirms that the increased myocardial ECV in the patients in our study was caused by diffuse myocardial fibrosis. Third, not all of the subjects in this study underwent computed tomography angiography or coronary angiography to exclude coronary heart disease, because invasive examination is not suitable for asymptomatic subjects. However, electrocardiography, magnetic resonance imaging cine, and delayed gadolinium enhancement imaging were performed to exclude overt coronary heart disease.

## Conclusions

T2DM patients with preserved EF show increased ECV but deteriorated GLS, which may be exacerbated by HT among these patients. HbA1c is an index that can independently predict T2DM patients’ LV myocardial deformation and tissue abnormalities.

## Data Availability

The datasets used and/or analysed during the current study are available from the corresponding author on reasonable request.

## Abbreviations

T2DM: Type 2 diabetes mellitus
LV: Left ventricular
ECV: Extracellular volume fraction
GLS: Global longitudinal strain
HF: Heart failure
HFpEF: HF with preserved ejection fraction
LVEF: Left ventricular ejection fraction
HT: Hypertension
CMR: Cardiac magnetic resonance
ECM: Extracellular matrix
FPG: Fasting plasma glucose
PG: Plasma glucose
OGTT: Oral glucose tolerance test
HbA1c: Hemoglobin A1c
SBP: Systolic blood pressure
DBP: Diastolic blood pressure
HCT: Hematocrit
GSP: Glycosylated serum protein
TC: Total cholesterol
F-CHO: Free cholesterol
TG: Triglycerides
HDL-C: High-density lipoprotein cholesterol
LDL-C: Low-density lipoprotein cholesterol
LGE: Late gadolinium enhancement
EDV: End-diastolic volume
ESV: End-systolic volume
LVM: LV mass
BSA: Body surface area
GRS: Global radial strain
GCS: Global circumferential strain
GLS: Global longitudinal strain
BMI: Body mass index
